# Emotional processing of sadness and disgust evoked by disaster scenes

**DOI:** 10.1002/brb3.2421

**Published:** 2021-11-22

**Authors:** Xin Wang, Jingna Jin, Wenbo Liu, Zhipeng Liu, Tao Yin

**Affiliations:** ^1^ Institute of Biomedical Engineering Chinese Academy of Medical Science & Peking Union Medical College Tianjin China; ^2^ Sinovation (Beijing) Medical Technology Co., Ltd; ^3^ Neuroscience Center Chinese Academy of Medical Science & Peking Union Medical College Beijing China

**Keywords:** disaster, disgust, emotional processing, sadness

## Abstract

**Objective:**

Disaster scenes produce long‐term negative feelings in those who experience them. Previous studies have focused on mitigating disaster impacts through directed forgetting or conscious suppression. However, the initial emotional processing of disaster scenes is not fully understood, hindering the comprehension of long‐term disaster impacts. This study aims to investigate how pictures of disaster scenes evoking disgust and sadness are processed via cortical electrical activity.

**Methods:**

Pictures of grief and mutilation from disasters were used to evoke sadness and disgust, respectively. Event‐related desynchronization (ERD) and event‐related potentials (ERPs) were used to quantify the intensity and time‐course of emotional processing.

**Results:**

The information processing of emotional pictures was stronger than neutral pictures, represented by greater declines of alpha ERD. In the posterior ERP components of N1 and EPN, amplitudes for emotional pictures were larger than those for neutral pictures, which reflected the effects of arousal on visual perception. In the anterior ERP components of P2, P3, and LPP, disgust pictures showed higher attention attraction and enhanced encoding memory processing.

**Conclusions:**

Disgust disaster scenarios induced long‐term prominent LPP, which may correspond with the long‐term negative impacts of the disaster.

## Graphical abstract

Disaster scenes produced long‐term negative feelings on experiencers. This study aimed to investigate how negative disaster scenes (disgust and sadness) were processed with ERD and ERPs. Results revealed that disgust disaster scenarios induced long‐term prominent LPP, which might imply the long‐term negative impacts of the disaster.

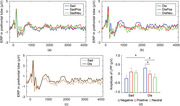

## 1 INTRODUCTION

Natural disasters and manmade accidents are common occurrences around the globe (Reifels, 2018; Roudini et al., 2017). Disasters not only cause death and economic losses but also leave severe negative emotions on those who experience them (Bond et al., 2020; Huang & Gan, 2018; Kannis‐Dymand et al., 2020; Reifels et al., 2013; Tang et al., 2017). Surveys show that disasters affect personal life and social communication for a long time, and may even cause mental illness (Berking & Wupperman, 2012; Geraerts & McNally, 2008; Kannis‐Dymand et al., 2020; Wang et al., 2016). In previous studies, directed forgetting and thought suppression paradigms were used to mitigate the negative emotions after disasters (Geraerts & McNally, 2008; Uzer & Brown, 2017). But the initial emotional processing of disaster scenes is still very lacking.

Across disasters, the most frequently reported negative emotions were fear and panic (Bond et al., 2020; Gruebner et al., 2018; Larson & Shin, 2018; Massazza et al., 2020). In fact, the sadness and disgust evoked by disaster scenes cannot be ignored. At disaster scenes, when individuals see others mourning relatives, they feel sadness themselves, which is triggered by empathy mechanisms (Morelli & Lieberman, 2013; Pawling et al., 2017). When individuals see bodily mutilation, they feel disgusted (Reifels et al., 2013). Several studies have shown the potential overlapping of fear, sadness, disgust, and anger (Bradley et al., 2001; Xu et al., 2017). In this study, we chose disaster scenarios with pure emotions as much as possible to investigate the emotional processing of sadness and disgust, which is of great significance for understanding the impacts of disaster on individuals.

Event‐related desynchronization (ERD) studies revealed that alpha‐band and lower beta‐band activity might reflect cortical activation associated with an initial arousing emotional stimulus (Meng et al., 2016; Schubring & Schupp, 2019). Event‐related potentials (ERPs) have enabled the assessment of neural responses to affective events with millisecond temporal resolution. For short latencies (100–200 ms), the P1 and N1 components are sensitive to physical stimulus factors (Olofsson et al., 2008). For middle latency (200–300 ms), the early posterior negativity (EPN) represents the selection of stimuli for further processing (Van Strien & Isbell, 2017). The anterior P2 has been reported to involve attention attraction in a bottom‐up manner (Lu et al., 2016). The frontal N2 ERPs reflect the regulation of automatic approach tendencies to positive stimuli (Ernst et al., 2013). For long latency (>300 ms), the P3 component, late positive component (LPC), and late positive potential (LPP) are associated with enhanced stimulus processing and memory encoding (Lu et al., 2016; Olofsson et al., 2008).

Studies have revealed that negative events and information evoke stronger physiological and emotional reactions compared to both neutral and positive events and information, a phenomenon called negativity bias (Liu et al., 2015; Olofsson et al., 2008; Schupp et al., 2004; van Hooff et al., 2014; Weinberg & Hajcak, 2010). At the early stage, the negativity bias framework emphasizes a rapid orienting of attention to facilitate processing efficiency and protect people from immediate danger and harm (Bar‐Haim et al., 2005; Liu et al., 2017; Miller & Martin, 2020; Olofsson et al., 2008; Torrence & Troup, 2018). The facilitated processing of attention at the early stage is mainly reflected by augmented amplitudes of posterior N1 and EPN (Olofsson et al., 2008; Weinberg & Hajcak, 2010) and anterior P2 (Lu et al., 2016). In contrast, the defensive motivation and “disease‐avoidance system” emphasizes attentional avoidance for negative stimuli, which suppresses sensory acquisition and stimulus perception (Koster et al., 2006; Lu et al., 2016; Oaten et al., 2009). When considering sadness and disgust evoked by disaster scenes, the effect of negative bias at the early stage is still not known. Thus, we asked: will the cortical electrical activity induced by disaster scenes appear as facilitated attention or suppressed attention? This question helps to direct investigation of the early information processing of negative disaster scenes.

Does the information processing of the disaster scene imply the negative impact of the disaster on the experiencer? This issue is also a focus of this study. The long latency ERP component reflects the enhanced stimulus processing and is thought to be closely related to memory encoding and cognition (Bond et al., 2020; Geraerts & McNally, 2008; Huang & Gan, 2018; Kannis‐Dymand et al., 2020; Lu et al., 2016; Olofsson et al., 2008). Pictures with high arousal or motivational significance have been shown to evoke larger amplitudes of LPC or LPP, such as erotic, mutilation, and threat images (Lu et al., 2016; Olofsson et al., 2008; Weinberg & Hajcak, 2010; Wheaton et al., 2013). When thinking about sadness and disgust evoked by disaster scenes, the enhanced stimulus processing at the later stage may also be an important clue for the long‐term psychological impacts of the disaster on experiences.

In this study, pictures of grief and mutilation in disaster scenes were used to evoke sadness and disgust respectively. The electroencephalogram (EEG) was recorded when participants viewed disaster scenes. ERD and ERPs were analyzed to evaluate the intensity of information processing and to follow the time course. We hypothesized that differentiated ERD or ERPs would be evoked by disgust and sadness disaster scenes, with differentiated information processing. This study will deepen the understanding of attention bias at the early stage and memory encoding at the later stage for negative disaster scenes.

## 2 MATERIALS AND METHODS

### 2.1 Volunteers

Twenty graduate students with a major in science and engineering (9 males, age: *M* = 27.5, *SD* = 3.1) participated in the experiments. All participants were right‐handed, native Chinese speakers, with normal or corrected‐to‐normal vision. None of them reported any mental illness or chronic use of prescription medication or alcohol within one year. All participants had provided informed consent before participating in the study. The participant characteristics were in accord with guidelines (Keil et al., 2014; Picton et al., 2000). The study was performed following the Declaration of Helsinki and was approved by the Ethics Committee of the Institute of Biomedical Engineering, Chinese Academy of Medical Sciences & Peking Union Medical College.

### 2.2 Materials

Pictures of grief in disaster scenes were used to evoke sadness, such as mourning relatives. Pictures of mutilation from disaster scenes were used to evoke disgust. Positive pictures of family reunion and beauty and neutral pictures of daily life scenes were chosen for comparison. In total, 180 pictures were collected, which were sourced from the International Affective Picture System (IAPS, RRID: SCR_016869) (Lang et al., 2008), Google, and Baidu photo library. All pictures were set at 512 × 378 pixels and 72 × 72 dpi. Pictures were divided into two groups. For the sadness group, 30 sadness, 30 positive, and 30 neutral scene pictures were selected and were referred to as “Sad,” “SadPos,” and “SadNeu.” For the disgust group, 30 disgust, 30 positive, and 30 neutral scene pictures were selected and were referred to as “Dis,” “DisPos,” and “DisNeu.” Positive and neutral scene pictures in the two groups were similar but not identical. Before the experiment, another 100 volunteers (48 males, age: *M* = 29.0, *SD* = 4.4) rated the valence and arousal of selected pictures according to the 9‐point self‐assessment manikin (SAM) (Bradley & Lang, 2007). Pictures were presented in a random order through E‐prime 2.0. For Sad and Dis pictures, the 100 volunteers were told to further note whether any negative emotion was evoked, such as sadness, disgust, anger, and fear.

### 2.3 Experimental protocols

The experiment consisted of two blocks, one for the sadness group and the other for the disgust group. For each participant, both blocks were performed. The block order was counterbalanced across participants. The time interval between the two blocks was 10 min. For each block, 90 trials were involved. Each trial was initiated by a small white fixation cross on the black computer screen, lasting for 4 s. The fixation cross was followed by the presentation of a random picture stimulus for another 4 s. Participants were informed to keep feeling the intensity of emotions as much as possible. When the picture disappeared, the participant judged the picture as negative, positive, or neutral by pressing “F,” “G,” or “H” on the keyboard. At the end of a block, participants reported their overall subjective feelings of the emotional pictures, such as no feeling at all, medium, strong, or intolerable.

### 2.4 Signal acquisition and processing

Experiments were conducted in a laboratory particularly suited to EEG acquisition, with soft light, good sound insulation, and suitable temperature and humidity. A 32‐lead ActiveTwo (Biosemi, Netherlands) was used for EEG acquisition. The EEG was recorded from 32 electrodes (FP1, FP2, AF3, AF4, F7, F5, F3, Fz, F4, F8, FC5, FC1, FC2, FC6, T7, C3, Cz, C4, T8, CP5, CP1, CP2, CP6, P7, P3, Pz, P4, P8, PO3, PO4, O1, Oz, O2) according to the international 10–20 system with a sampling rate of 1024 Hz. The vertical electrooculogram (EOG) was recorded infraorbitally at the left eye. The horizontal EOG was recorded lateraorbitally of the right eye. All electrode impedances were maintained below 5 kΩ.

Data processing was performed using MATLAB (version 2018). Band‐pass filtering of 0.1–60 Hz was applied to remove the high‐frequency interference and low‐frequency drift in EEG. A notch filter at 50 Hz was used to eliminate power‐line interference. The EEG of 32 leads was averaged as the reference for data conversion. Independent component analysis (ICA) was used to eliminate interference from eye movements in the EEG.

A time–frequency diagram based on the continuous wavelet transform (CWT) was used to visually display power changes in both the time and frequency domains. The CWT provided the decomposition of the signal *x*(*t*) onto a set of basic functions that were obtained by dilation of a “mother” wavelet *ψ*(*t*), provided in formula (1), where the continuous variables *a* and *b* were the scale and translation parameters, respectively. The mother wavelet *ψ*(*t*) was set as a complex Morlet wavelet. The power density was calculated using formula (2).

(1) $$CWT_{x}\left(a,b\right)=\frac{1}{\sqrt{\left|a\right|}}\int_{-\infty }^{+\infty }X(t)\;\Psi \;\left(\frac{t-b}{a}\right)\;\;dt,$$

(2) $$P_{X}\left(a,b\right)={\left|CWT_{X}\;\left(a,b\right)\;\right|}^{2}\;.$$

The ERD was used to quantify the time–frequency diagrams and was defined as the decrease in power in a specific frequency band during a test interval compared to a reference interval. The ERD was calculated using formula (3), where ∆*i* and ∆*re* represent the test interval and the reference interval, respectively. *P*_∆_ *_i_* and *P*_∆_ *_re_* represent the power of the signal in the corresponding interval, which was computed by adding up the square values of voltage levels included in the considered time segment. *D_i_* was the quantification of ERD, quantified as the intensity of information processing.

(3) $$D_{i}=\left(P_{\Delta i}-P_{\Delta re}\right)/P_{\Delta re}.$$

ERPs were used to follow the time course and details of information processing in the millisecond‐range resolution. Based on behaviorally correct trials, EEG signals induced by pictures of the same condition were averaged. The mean amplitude of the EEG signal during the 200 ms before the picture appeared was calculated as the baseline for calibration. ERPs were obtained using formula (4), where *t* = −200 to 4000 ms, representing the time interval from 200 ms before the picture appeared to 4000 ms after the picture appeared; *x*(*t*) was the EEG signal. The N1 and EPN were scored as the mean activity on the occipital and parietooccipital electrodes (PO3, PO4, O1, Oz, O2), at 100–200 ms and 270–470 ms respectively. The P2, N2, P3, and LPP were scored as the mean activity on the prefrontal electrodes (Fp1, Fp2, AF3, AF4), at 130–190, 200–250, 250–380, and 700–4000 ms, respectively.

(4) $$E\left(t\right)=X\left(t\right)-\frac{1}{200}\sum_{j=-200}^{0}X(j).$$

### 2.5 Statistical analysis

One‐way ANOVA was used for comparison among broad categories (negative, positive, and neutral). Two‐way ANOVA was used for comparison among emotional content (Sad, SadPos, SadNeu, Dis, DisPos, and DisNeu). Post hoc testing of significant main effects was conducted using a Bonferroni correction. Due to our prior hypotheses, the pairwise comparison between sadness and disgust and fixed comparisons among pictures were further analyzed. Partial *η* ^2^ was calculated to estimate the effect size of ANOVA and Cohen's *d* was calculated to estimate the effect size of pairwise comparison. All statistical analysis was performed using SPSS software (version 21.0). According to the guidelines (Clayson et al., 2019; Keil et al., 2014; Pernet et al., 2020), the statistical power analysis was performed using G*Power 3.1.9.7; results are shown in supplementary materials. Power analysis was not performed for the main effect and nor for interaction of ANOVA that were not statistically significant.

## 3 RESULTS

### 3.1 Behavioral results

The valence and arousal ratings of the Sad, Dis, Pos (SadPos, DisPos), and Neu (SadNeu and DisNeu) are shown in Table 1. One‐way ANOVA analysis showed that there were significant differences in valence and arousal among Dis, Sad, Neu, and Pos (both *ps* < .001), and post hoc tests showed significant differences between any two of them (All *ps* < .001). The probability to evoke specific negative emotion was calculated. For Sad pictures, the average probability to evoke sadness was 95.5%. For Dis pictures, the average probability to evoke disgust was 97.1%.

**TABLE 1:** The valence and arousal ratings of pictures (mean ± SD)

Picture type	Valence	Arousal
Sad	2.62 ± 0.28	6.30 ± 0.39
Dis	1.33 ± 0.19	8.48 ± 0.22
SadPos, DisPos	6.60 ± 0.34	5.81 ± 0.29
SadNeu, DisPos	4.84 ± 0.28	2.97 ± 0.26

The accuracy of emotion judgment was quantified. Six participants identified one of the neutral pictures as positive, and the accuracy of the neutral pictures was 99.5%. Two participants identified one positive image as neutral, and the accuracy of positive pictures was 99.8%. For negative pictures, the accuracy was 100%. No significant statistical difference was found among the accuracy of emotion judgment.

Participants' self‐reports showed that disgust‐category disaster pictures caused much stronger feelings than other pictures.

### 3.2 ERD results

**FIGURE 1:**
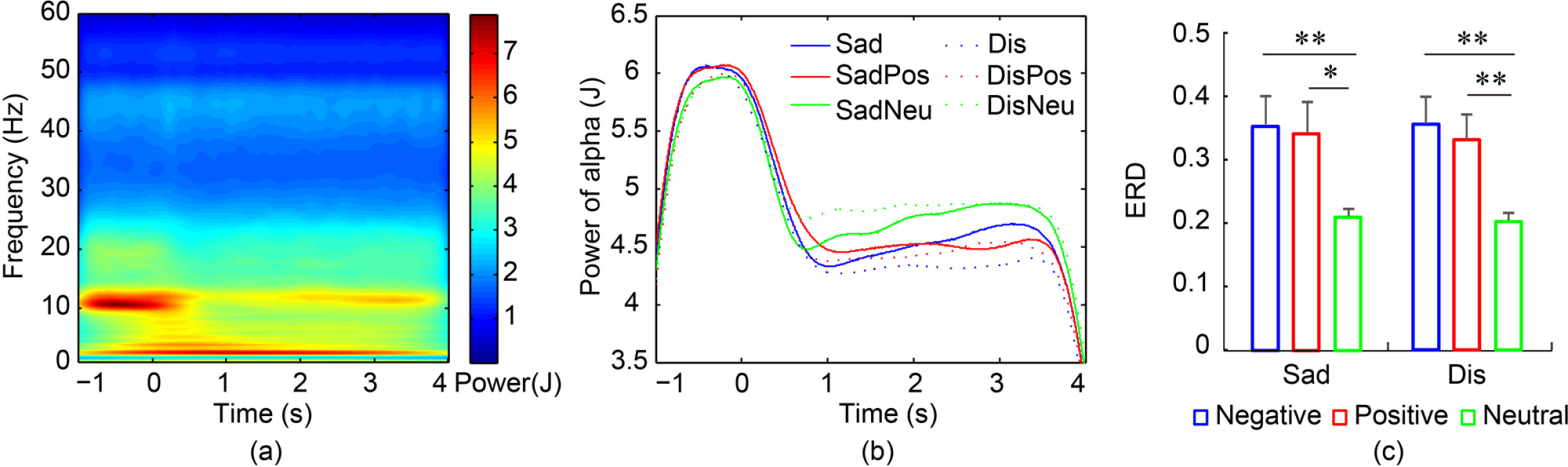
Time–frequency analysis of ERD. (a) Time–frequency diagram. (b) Alpha power declines. (c) Comparison of alpha ERD. ERD of emotional pictures was significantly larger than neutral pictures. ^*^ *p* < .05, ^**^ *p* < .01

The average wavelet time–frequency diagram for all leads from all subjects when Sad pictures were presented is shown in Figure 1a. The power before the picture appeared was mainly concentrated in the low‐frequency band, especially alpha (8–13 Hz). When the picture was presented, the power of the alpha band decreased sharply and reached its minimum value at approximately 1 s. In the later stages (2–4 s), the power of the alpha band showed a trend of gradual recovery. The decline in alpha power has been called “alpha blocking,” and reflects an increased excitability level of neurons in the involved cortical areas.

Time–frequency diagrams for the other pictures were similar to those of the Sad pictures. To compare power changes among different pictures in detail, the power of the alpha band was extracted. As shown in Figure 1b, alpha power was similar among different pictures during –1 to 0 s. However, during 0−1 s, the alpha power of emotional pictures decreased much further than that of neutral pictures.

ERD was used to quantify the differences in the power decreases among the different pictures. In this study, the ERD of the alpha band was calculated, with the test interval and the reference interval set at 1 to 2 and −0.75 to –0.25 s, respectively, based on the time–frequency diagram. Two‐way ANOVA comparison of ERD showed significant differences among negative, positive, and neutral pictures (*F*(2,114) = 9, *p* < .001, partial *η* ^2^ = 0.22), no differences between sadness and disgust groups (*F*(1,114) = 0.02, *p* = .879, partial *η* ^2^ < 0.01), and no interaction effect (*F*(2,114) = 0.01, *p* = .988, partial *η* ^2^ <0.01). Post hoc tests showed that the ERD of Sad (*p* = .009, Cohen's *d* = 1.23) and SadPos (*p* = .020, Cohen's *d* = 1.11) pictures were both significantly larger than that of SadNeu pictures, as shown in Figure 1c. Similarly, the ERD of Dis (*p* = .004, Cohen's *d* = 1.32) and DisPos (*p* = .009, Cohen's *d* = 1.17) pictures were also significantly larger than that of the DisNeu pictures. It can be seen that the information processing for emotional pictures is more active than information processing for neutral pictures.

**FIGURE 2:**
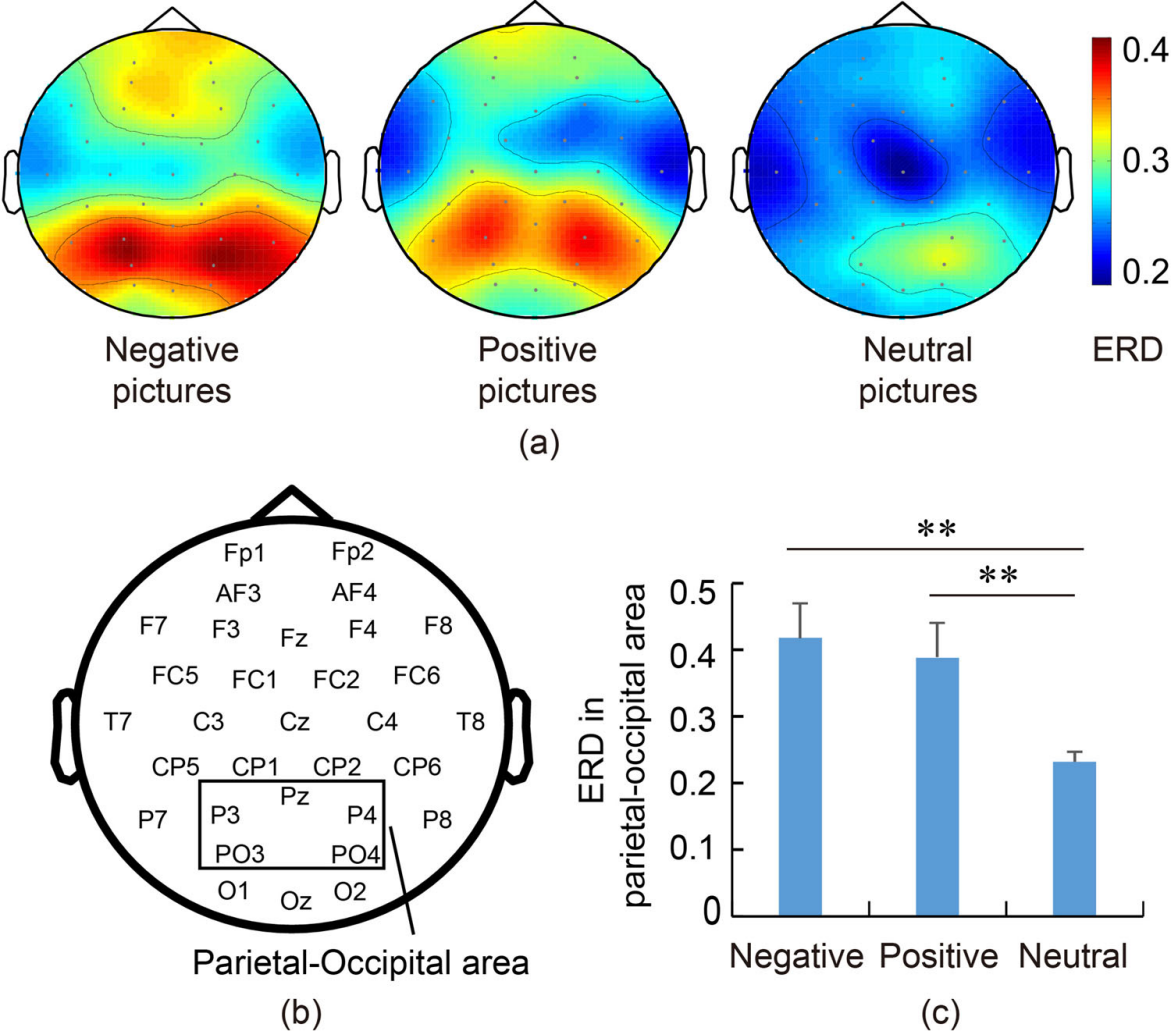
Spatial analysis of ERD. (a) Spatial distribution of ERD. (b) Leads in parietal‐occipital area. (c) Comparation of ERD in parietal–occipital area. In the parietal–occipital area, ERD for emotional pictures was significantly larger than that for positive or neutral pictures. ^**^ *p* < .01

To explore the cortical distribution of ERD, topographic maps were drawn based on ERD, as shown in Figure 2a. Overall, the posterior cortex was highlighted among the emotional pictures. The ERD in the parietal–occipital lobe was calculated and the differences among pictures were significant (*F*(2,117) = 5.17, *p* = .011, partial *η* ^2^ = 0.24). Post hoc tests showed that the ERD for negative pictures (*p* = .003, Cohen's *d* = 1.37) and positive pictures (*p* = .008, Cohen's *d* = 1.18) were both significantly higher than that for neutral pictures, which reflected much more active information processing for emotional pictures.

### 3.3 Posterior N1 and EPN components

**FIGURE 3:**
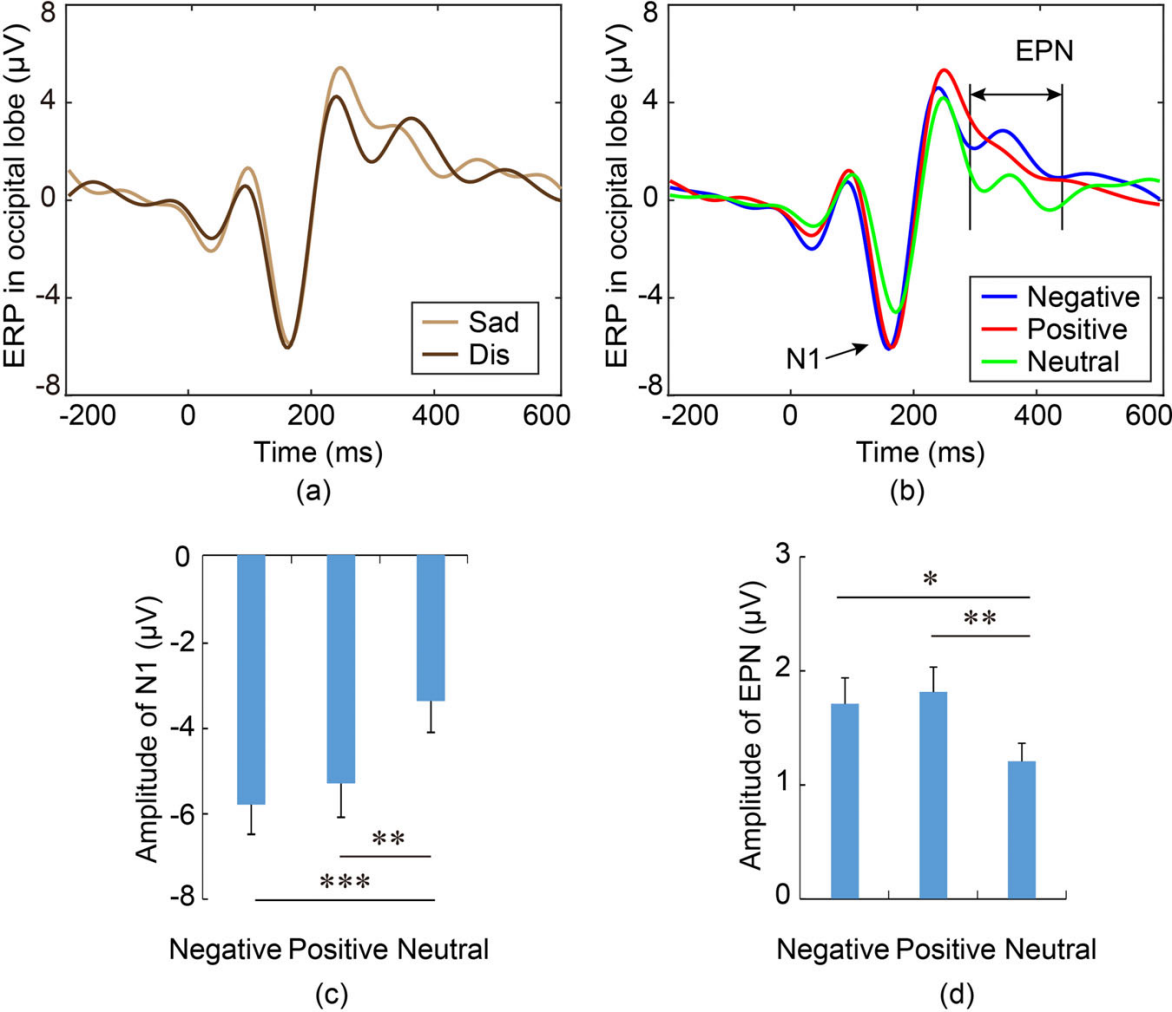
Posterior N1 and EPN components. (a) ERP of Sad and Dis pictures. (b) ERP of negative, positive, and neutral pictures. (c), (d) Amplitude comparison of N1 and EPN. The larger amplitudes of N1 and EPN components in the occipital lobe for emotional pictures reflected the arousal effects on visual perception. ^*^ *p* < .05, ^**^ *p* < .01, ^***^ *p* < .001.

ERPs in the occipital lobe mainly reflected the visual perception of emotional pictures. First, we compared the disgust and sadness images with the early ERPs in the occipital lobe, and the results showed no obvious difference, as shown in Figure 3a. Therefore, the ERPs induced by negative, positive, and neutral images of the two groups were merged respectively to explore the visual perception of negative pictures.

As shown in Figure 3b, two components showed differences between emotional pictures and neutral pictures, namely N1 and EPN. One‐way ANOVA statistical comparisons showed significant differences among pictures for N1 (*F*(2,117) = 3.48, *p* = .035, partial *η* ^2^ = 0.0765) and EPN (*F*(2,117) = 3.39, *p* = .038, partial *η* ^2^ = 0.0731). Post hoc tests showed that the amplitudes of N1 for emotional pictures were significantly larger than that for neutral pictures (*p* < .001, Cohen's *d* = 0.69 for negative vs. neutral pictures, *p* = .002, Cohen's *d* = 0.59 for positive vs. neutral pictures). During 270–470 ms, the EPN was significantly more prominent for emotional pictures than that for neutral pictures (*p* = .006, Cohen's *d* = 0.68 for positive vs. neutral pictures, *p* = .014, Cohen's *d* = 0.57 for negative vs. neutral pictures). The larger amplitudes of N1 and EPN components in the occipital lobe for emotional pictures reflected the effects of arousal on visual perception.

### 3.4 Anterior P2, N2, and P3 components

**FIGURE 4:**
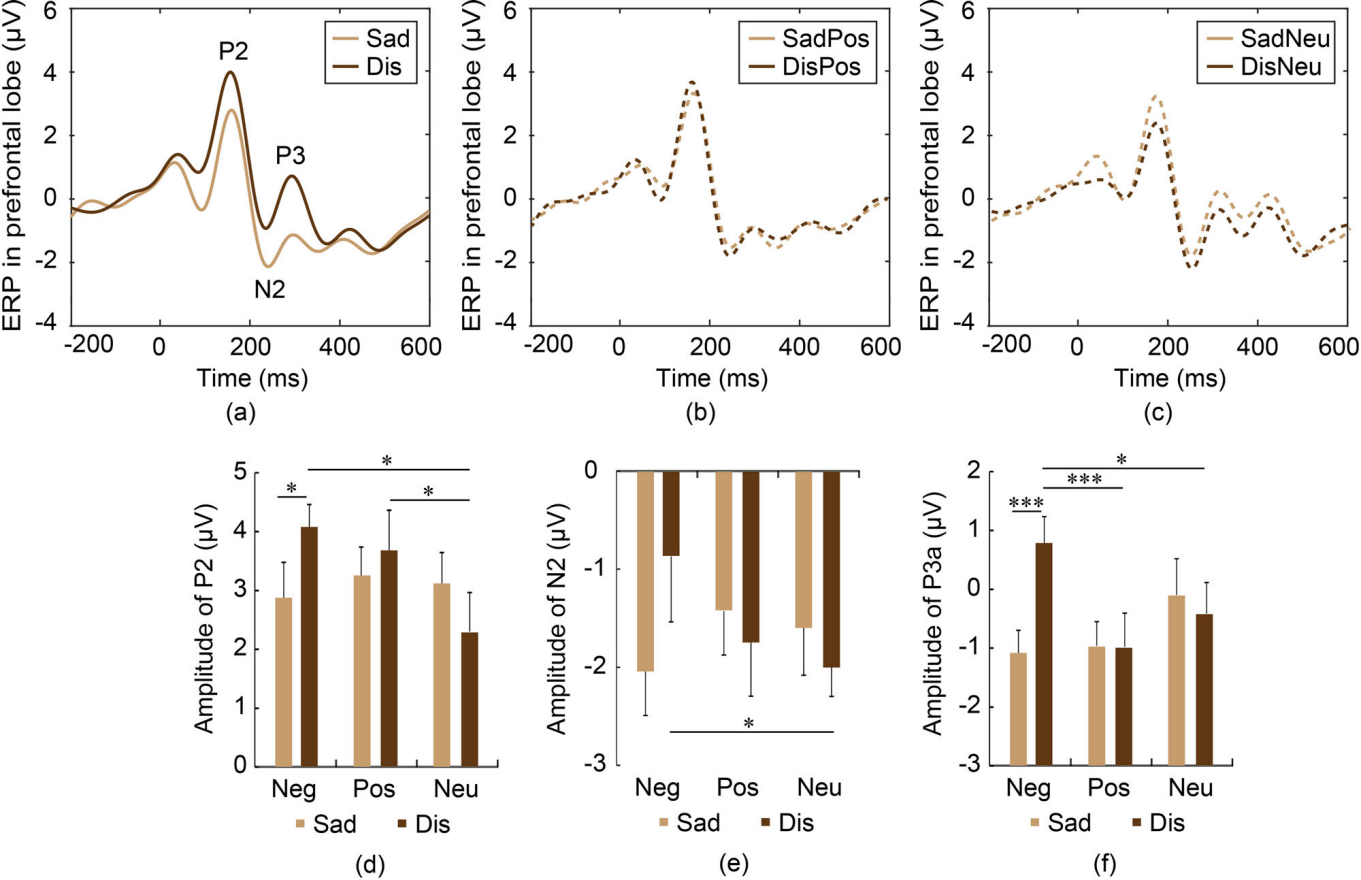
ERPs in the prefrontal lobe. (a) ERPs of Sad and Dis pictures, (b) ERPs of SadPos and DisPos pictures, (c) ERPs of SadNeu and DisNeu pictures, (d)–(f) Amplitude comparisons of P2, N2, P3. For disgust pictures, the emotional brain activity was much stronger and the automatic approach tendency was weaker than that for other pictures. ^*^ *p* < .05, ^***^ *p* < .001

Three ERP components in the prefrontal lobe were analyzed, P2, N2, and P3. As shown in Figure 4a, the potential of Dis pictures was higher than that of Sad pictures. For positive and neutral pictures, the potentials did not show any difference between the two groups, as shown in Figure 4b and c.

Two‐way ANOVA showed no difference among pictures, or between groups and no interaction effect for all components in the prefrontal lobe. Post hoc tests showed that the P2 amplitude of Dis pictures was significantly higher than that of Sad pictures (*p* = .0341, Cohen's *d* = 0.61), as shown in Figure 4d. In addition, the P2 amplitude of Dis (*p* = .0157, Cohen's *d* = 0.71) and DisPos (*p* = .0112, Cohen's *d* = 0.75) pictures were significantly higher than those of DisNeu. The N2 amplitude of Dis pictures was significantly lower than that of DisNeu pictures (*p* = .0463, Cohen's *d* = 0.56), as shown in Figure 4e. The lower amplitude of N2 for disgust pictures in the prefrontal lobe might reflect a weaker approach tendency. For P3, the amplitude of Dis pictures was significantly greater than that of Sad (*p* < .001, Cohen's *d* = 1.35), DisPos (*p* < .001, Cohen's *d* = 1.27), as well as DisNeu (*p* = .0489, Cohen's *d* = 0.56), as shown in Figure 4f.

### 3.5 Anterior LPP component

**FIGURE 5:**
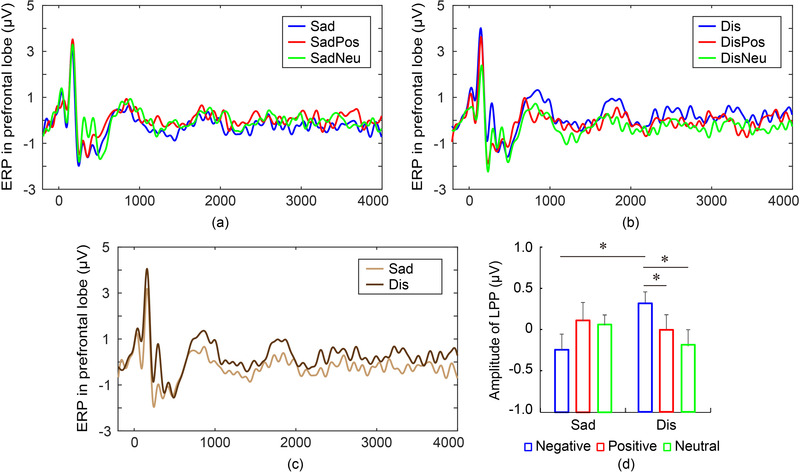
Long‐term ERPs. (a) Long‐term ERPs for Sad, SadPos and SadNeu pictures. (b) Long‐term ERPs for Dis, DisPos and DisNeu pictures. (c) Long‐term ERPs for Sad and Dis pictures. (d) Amplitude comparison of LPP. The amplitude of LPP for Dis pictures was significantly larger than that for Sad pictures and other pictures. ^*^ *p* < .05.

To further investigate the information processing of disgust pictures, the long‐term ERPs were compared among pictures. Two‐way ANOVA showed no main effects of LPP among pictures and between groups, and a small interaction effect (*F*(2,114) = 2.480, *p* = .090, partial *η* ^2^ = 0.06). As shown in Figure 5a, there was no obvious difference among Sad, SadPos, and SadNeu pictures. In the disgust group, post hoc tests showed that the LPP amplitudes of Dis pictures were significantly greater than those of DisPos pictures (*p* = .0439, Cohen's *d* = 0.57) or DisNeu pictures (*p* = .0408, Cohen's *d* = 0.58), as shown in Figure 5b and d. Moreover, the LPP amplitude of Dis pictures was significantly greater than that of Sad pictures (*p* = .0224, Cohen's *d* = 0.66), as shown in Figure 5c and d. The larger LPP amplitude of Dis pictures reflected stronger emotional perception, which lasted up to 4 s. The prominent LPP components for disgust pictures imply enhanced encoding memory processing and impaired disengagement.

## 4 DISCUSSION

In this study, we aimed to explore the emotional processing of disgust and sadness evoked by disaster scenes. From the ERD analysis, emotional pictures showed much more active information processing than neutral pictures. In the posterior ERP components of N1 and EPN, amplitudes for emotional pictures were larger than that for neutral pictures. In anterior ERP components, amplitudes of P2, P3, and LPP for disgust pictures were larger than that for other pictures, but the amplitude of N2 was lower than other pictures. Specific ERP performance for disgust may reflect specific information processing. For sadness, no prominent electrical cortical activity was observed.

### 4.1 Information processing based on alpha ERD

In the present study, alpha‐band power showed a sharp decrease in brain activity when participants viewing pictures. Several studies in the cognitive domain (i.e., perception, attention, working memory) have also revealed a decrease in alpha power in brain regions activated during a cognitive task (Foxe & Snyder, 2011; Klimesch, 2012; Mathewson et al., 2011). While in a state of conscious rest, the alpha activity was obvious (Onoda et al., 2007). The temporal pattern of alpha ERD was similar to that observed in the default mode network (DMN). The DMN is composed of several brain regions that are connected to maintain healthy metabolic activities, and are very active when the brain is at rest. The DMN appears to be deactivated or inhibited when the brain is performing tasks (Chen et al., 2008). Thus, we believe that both the ERD of alpha rhythm and the deactivation of the DMN reflect the activity of the brain in performing tasks and the intensity of information processing of external stimuli.

In this study, the alpha ERD of emotional pictures was significantly stronger than that of neutral pictures, indicating more intense information processing for emotional pictures. Previous studies reported a negative association of the power in the alpha band with increasing stimulus arousal (De Cesarei & Codispoti, 2011; Schubring & Schupp, 2019). Our result was consistent with existing studies. Moreover, this study revealed that the alpha ERD mainly occurred in 0–1 s and was prominent in the parietal‐occipital lobe, suggesting the importance of visual perception in emotional picture processing.

### 4.2 Early visual perception based on posterior N1 and EPN

As an external visual stimulus, emotional pictures activated the occipital lobe for visual processing in the early stage. Studies have argued that negative events and information evoke stronger physiological and emotional reactions compared to both neutral and positive events and information (Olofsson et al., 2008; Weinberg & Hajcak, 2010). The negativity bias framework emphasizes the facilitated processing of attention at the early stage, reflected as augmented amplitudes of posterior P1, N1, and EPN (Olofsson et al., 2008; Weinberg & Hajcak, 2010) and anterior P2 (Lu et al., 2016). Some studies have suggested that disgust facilitates a disease‐avoidance mechanism, reflected as smaller occipital P1 than invalidly cued targets in a dot‐probe task (Liu et al., 2015; Oaten et al., 2009). van Hooff et al. (2014) revealed that attention was temporarily grabbed when disgust‐evoking pictures were presented for 100 and 200 ms, but not for 500 or 800 ms (van Hooff et al., 2014). It is speculated that the duration of image presentation may be a factor affecting attentional capture. In this study, pictures were presented over 4000 ms and the attention was not attracted quickly.

Consistent with existing research, the EPN consisted of a positive‐going waveform over temporooccipital sites (Olofsson et al., 2008). The main theoretical interpretation of the EPN is that it indexes “natural selective attention,” which selects affectively arousing stimuli for further processing (Olofsson et al., 2008). The EPN is also associated with the functioning of the basic motivational systems of approach and avoidance, reflected as augmented amplitude for stimuli of evolutionary significance, such as a threatening snake. In this study, the amplitudes of N1 and EPN components in the occipital lobe were much larger for emotional pictures than that for neutral pictures, which reflected the effect of arousal on visual perception.

### 4.3 Middle attention processing based on anterior P2 and N2

P2 is an early component that is modulated by automatic emotional processing (Lu et al., 2016). The amplitude of the anterior P2 has been reported to increase when a stimulus attracts attention in a bottom‐up manner. A previous study showed that disgusting non‐face pictures produced higher P2 amplitudes than neutral pictures (Lu et al., 2016). The present study showed that disgusting disaster scenes produced higher P2 amplitudes than neutral pictures, theoretically attracting more attention. Moreover, the P2 amplitude of disgusting disaster scenes was larger than the sadness disaster scene. This extra early attention could be important for later memory processes and future stimulus avoidance (Lu et al., 2016).

N2 is involved in attentional processes associated with the detection of stimulus novelty (Lu et al., 2016). At the preconscious level, as more stimulus features are detected, the brain begins to integrate these features into meanings. Previous approach‐avoidance studies revealed that people tended to approach positive factors and avoid negative factors automatically, reflected by as larger N2 amplitudes for positive factors and lower N2 amplitudes for negative factors (Ernst et al., 2013; Liu et al., 2015). In this study, the amplitudes of N2 for Dis pictures were both lower than that of Sad pictures, positive pictures, and neutral pictures. For Dis pictures, the contents were so negative that they might induce a weaker approach tendency, which impeded searches for novel and salient information.

### 4.4 Late emotional perception based on anterior P3 and LPP

In this study, the amplitudes of P3 for Dis pictures were both greater than those of Sad pictures, positive pictures, and neutral pictures. Major determinants of P3 amplitude were task‐relevance, motivational significance, arousal level, and the influence of these factors on mental resource allocation (Olofsson et al., 2008). Reviews have proposed that P3 reflects dopaminergic modulatory effects exerted by the phasic activity of the locus coeruleus–norepinephrine system (Olofsson et al., 2008).

In previous studies, long‐lasting (400–1000 ms) elevated ERP positivity to arousing pictures was a common finding (Lee et al., 2017; Olofsson et al., 2008; Weinberg & Hajcak, 2010). Evidence suggested that the slow‐wave (400–1000 ms) was involved in memory evaluation and memory encoding (Lu et al., 2016; Olofsson et al., 2008). Evolutionary models suggest that disgust pictures feature high motivational salience. In this study, the amplitudes of long‐term LPP (700–4000 ms) for Dis pictures were greater than that of Sad pictures, positive pictures, and neutral pictures. The prominent long‐term LPP amplitude for Dis pictures implies enhanced encoding processing of memory, which could be a reason for the long‐term negative impacts of disasters.

Studies have reported that negative information was hard to disengage, manifested as longer responses in cognitive tasks (Baik et al., 2018; Toffanin et al., 2011; Zhao et al., 2019). In this study, the larger P3 and long‐term LPP amplitudes for Dis pictures may also reflect the impaired disengagement. The response to the disgust disaster scenario does not return to baseline for a long time, which may also be a reason for the long‐term negative impacts of disasters.

### 4.5 Deconstructing negative emotions of specific picture content

Currently, several studies have deconstructed negative emotions based on specific picture content. Miller and Martin (2020) demonstrated that the late event‐related potential positivity was influenced by social relevance (the P3b) or the level of aggression displayed by the stimulus (the LPC). Weinberg et al. examined the time‐course of electrocortical processing of emotional stimuli—both as a function of broad affective categories (i.e., pleasant, neutral, and unpleasant) and more specific content‐based categories of pictures (Weinberg & Hajcak, 2010). Results showed that erotic‐ (e.g., nude couples) and mutilation‐themed images (e.g., bomb victims) elicited the largest LPPs within pleasant and unpleasant pictures, respectively (Weinberg & Hajcak, 2010). Evolutionary models suggest that mutilation images that evoke disgust should be high in motivational salience (Wheaton et al., 2013). Results showed that disgusting and threatening images elicited equivalent LPPs (Wheaton et al., 2013). Lu et al. revealed that extremely disgusting stimuli elicited a more prominent late positive component than moderately disgusting stimuli (Lu et al., 2016). These studies confirm the importance of specific picture content in exploring electrical cortex processing, beyond arousal and valence.

In this study, mutilation pictures from disaster scenes evoked more prominent long‐term (lasting for 4000 ms) LPP than all other pictures, which had not been seen in the existing studies. Moreover, the participants' self‐reports showed that disgust disaster pictures evoked much stronger impacts than other pictures. Thus, we speculate that disgust disaster pictures might evoke specific information processing. With specific emotional experience and specific brain activity, the results in this study may be helpful in understanding the long‐term negative impacts of disasters (Lench et al., 2013; Lench et al., 2011; Lindquist et al., 2013).

### 4.6 Behavioral performance of emotion judgment

According to the density hypothesis, positive information is more similar than negative information; this is a true property of information ecology (Alves et al., 2017, 2017a, 2019; Koch et al., 2016). The density hypothesis assumes that positive information is processed more quickly than negative information and has been shown to recognize positive words and pictures more quickly and accurately than negative words and pictures (Unkelbach et al., 2008; Unkelbach et al., 2010; Wu et al., 2019, 2020). However, according to the evolutionary models, disgust and fear evoking stimuli feature high motivational salience and the negativity bias framework emphasizes a rapid orienting of attention to facilitate processing efficiency and protect people from immediate danger and harm (Bar‐Haim et al., 2005; Liu et al., 2017; Miller & Martin, 2020; Olofsson et al., 2008; Torrence & Troup, 2018). Which stimulus is processed more quickly and accurately may have to be analyzed by specific experimental protocols. In two studies (Wu et al., 2019, 2020), the picture disappeared before the participants made a response and the time for presenting pictures was less than 1 s. In this study, each picture was presented for 4 s, which was far longer than the action time for valence judgment. Therefore, the behavioral performance could not indicate whether positive or negative pictures were processed more quickly. Moreover, the valence was determined when the picture disappeared. Since the pictures were presented long enough, the accuracies were all above 99% and no significant statistical difference was found between negative and positive pictures.

### 4.7 Guidance for regulation of disgust evoked by disaster scenes

Stronger long‐term negative emotional processing for disgust disaster scenarios may cause more severe psychological trauma to the individuals. This finding may be a clue to reducing disaster impact. This study will deepen the understanding of negative scenarios at disaster sites and promote the understanding of post‐traumatic stress disorder (PTSD). To regulate disgust evoked by disaster scenes, more conscious efforts and positive elements will be needed.

## 5 CONCLUSION

This study investigated the information processing of disgust and sadness evoked by disaster pictures. The power spectrum showed that the alpha ERD of emotional pictures was stronger than neutral pictures, indicating more intense information processing for emotional pictures. In the posterior ERP components of N1 and EPN, amplitudes for emotional pictures were larger than that for neutral pictures, which reflected the effects of arousal on visual perception. In the anterior ERP components of P2, P3, and LPP, disgust pictures showed higher attention attraction and enhanced encoding memory processing. The long‐term prominent LPP for disgust may have implications for the long‐term negative impacts of a disaster.

## FUNDING

National Key Research and Development Program of China, Grant Number: 2018YFC0115600; National Natural Science Foundation of China, Grant Numbers: 52107241, 52007199; Natural Science Foundation of Tianjin, Grant Number: 19JCYBJC28900.

## CONFLICT OF INTEREST

The authors declare that they have no conflict of interest.

### PEER REVIEW

The peer review history for this article is available at https://publons.com/publon/10.1002/brb3.2421

## AUTHOR CONTRIBUTIONS

Xin Wang and Tao Yin conceived and designed the experiments. Xin Wang and Jingna Jin performed the experiments and analyzed the data. Xin Wang, Wenbo Liu, and Zhipeng Liu wrote and revised the manuscript. Zhipeng Liu and Tao Yin were responsible for critically revising the article for important intellectual content involved in the article and approved the final version of the article for publication.

## Supporting information

Supporting information

## Data Availability

The data of this study will be available on request to the corresponding author.

## APPENDIX A

### A.1

IAPS pictures in each picture category were as follows:

Positive pictures: 2091, 2216, 2303, 2304, 2310, 2311, 2320, 2340, 2341, 2344, 2345, 2360, 2370, 2381, 2389, 2391, 2540, 2560, 2650, 2655, 2660, 2890, 2900.2, 4100, 4150, 4598, 4599, 4601, 4603, 4605, 4606, 4609, 4610, 4614, 4640, 4641, 4700, 8330, 8460, 8461, 9070.

Neutral pictures: 2200, 2210, 2214, 2215, 2240, 2250, 2270, 2440, 7000, 7002, 7004, 7006, 7009, 7010, 7020, 7025, 7031, 7035, 7040, 7050, 7060, 7080, 7090, 7095, 7150, 7160, 7170, 7175, 7182, 7183, 7190, 7200, 7205, 7211, 7217, 7220, 7224, 7234, 7235, 7490, 7491, 7700, 7705.

Disgust pictures: 3000, 3010, 3030, 3051, 3060, 3061, 3062, 3063, 3064, 3071, 3080, 3130, 3150, 3168, 9253.

Sadness pictures: 2141, 230, 2800, 2900, 2900.1, 9250.

Nineteen positive pictures, 17 neutral pictures, 15 disgust pictures, and 24 sadness pictures were sourced from public resources on the Internet, including Google photo library (https://www.google.com/) and Baidu (https://www.baidu.com/). The principle for collecting pictures from public sources is that the picture was sourced from a disaster scene and the content is similar to the IAPS pictures. Positive pictures of family reunion and beauty, neutral pictures of daily life scenes, disgust pictures of mutilation in disaster scenes, and sadness pictures of crying for relatives leaving in disaster scenes were collected.
