# Supplementary material for: Emotional processing of sadness and disgust evoked by disaster scenes
Source: Brain Behav. 2021 Nov 22;11(12):e2421. doi: 10.1002/brb3.2421 (PMC8671793; doi:10.1002/brb3.2421)
Supplement: Supplementary file 1 — Supporting information [file BRB3-11-e2421-s001.docx]

Supplementary materials

Supplementary Table 1: Statistical power analysis of ERD (Fig.1(c))

|  | Among negative, positive, and neutral pictures | Sad vs. SadNeu | SadPos vs. SadNeu | Dis vs. DisNeu | DisPos vs. DisNeu |
| --- | --- | --- | --- | --- | --- |
| *p* | <0.001 | 0.009 | 0.020 | 0.004 | 0.009 |
| partial *η^2^* | 0.22 | - | - | - | - |
| Cohen’s d | - | 1.23 | 1.11 | 1.32 | 1.17 |
| Statistical power | 0.91 | 0.92 | 0.91 | 0.91 | 0.88 |

Supplementary Table 2: Statistical power analysis of ERD (Fig.2(c))

|  | Among negative, positive, and neutral pictures | Negative vs. Neutral | Positive vs. Neutral |
| --- | --- | --- | --- |
| *p* | 0.011 | 0.003 | 0.008 |
| partial *η^2^* | 0.24 | - | - |
| Cohen’s d | - | 1.37 | 1.18 |
| Statistical power | 0.94 | >0.99 | >0.99 |

Supplementary Table 3: Statistical power analysis of early posterior N1 (Fig.3(c))

|  | Among negative, positive, and neutral pictures | Negative vs. Neutral | Positive vs. Neutral |
| --- | --- | --- | --- |
| *p* | 0.035 | <0.001 | 0.002 |
| partial *η^2^* | 0.0765 | - | - |
| Cohen’s d | - | 0.69 | 0.59 |
| Statistical power | 0.95 | 0.84 | 0.74 |

Supplementary Table 4: Statistical power analysis of early posterior EPN (Fig.3(d))

|  | Among negative, positive, and neutral pictures | Negative vs. Neutral | Positive vs. Neutral |
| --- | --- | --- | --- |
| *p* | 0.038 | 0.006 | 0.014 |
| partial *η^2^* | 0.0731 | - | - |
| Cohen’s d | - | 0.68 | 0.57 |
| Statistical power | 0.95 | 0.95 | 0.90 |

Supplementary Table 5: Statistical power analysis of anterior P2, N2, and P3

|  | Fig. 4(d) | | | Fig. 4(e) | Fig. 4(f) | | |  |
| --- | --- | --- | --- | --- | --- | --- | --- | --- |
|  | Dis vs. Sad | Dis  vs. DisNeu | DisPos vs. DisNeu | Dis  vs. DisNeu | Dis  vs.  Sad | Dis  vs. DisPos | Dis  vs. DisNeu | |
| *p* | 0.034 | 0.016 | 0.011 | 0.046 | <0.001 | <0.001 | 0.049 | |
| Cohen’s d | 0.61 | 0.71 | 0.75 | 0.56 | 1.35 | 1.27 | 0.56 | |
| Statistical power | 0.78 | 0.80 | 0.80 | 0.77 | 0.98 | 0.97 | 0.78 | |

Supplementary Table 6: Statistical power analysis of LPP

|  | Fig. 5(d) | | |
| --- | --- | --- | --- |
|  | Dis vs. DisPos | Dis vs. DisNeu | Dis vs. Sad |
| *p* | 0.0439 | 0.0408 | 0.0224 |
| Cohen’s d | 0.57 | 0.58 | 0.66 |
| Statistical power | 0.77 | 0.77 | 0.78 |

| Supplementary Table 7: The valence and arousal scores of pictures (mean ± SD) | | |
| --- | --- | --- |
| Picture type | Valence | Arousal |
| Sad | 2.62±0.28 | 6.30±0.39 |
| Dis | 1.33±0.19 | 8.48±0.22 |
| SadPos | 6.53±0.42 | 5.78±0.35 |
| DisPos | 6.67±0.22 | 5.85±0.21 |
| SadNeu | 4.84±0.28 | 2.98±0.31 |
| DisNeu | 4.89±0.20 | 2.97±0.20 |

| Supplementary Table 8: The probability to evoke negative emotions (%) | | | | |  |
| --- | --- | --- | --- | --- | --- |
| Picture type | Sadness | Disgust | Anger | Fear | |
| Sad | 95.5% | 3.2% | 2.5% | 2.8% | |
| Dis | 5.3% | 97.1% | 4.1% | 11.0% | |
